# The natriuretic peptides system in the pathophysiology of heart failure: from molecular basis to treatment

**DOI:** 10.1042/CS20150469

**Published:** 2015-12-04

**Authors:** Massimo Volpe, Marino Carnovali, Vittoria Mastromarino

**Affiliations:** * Cardiology Department, Clinical and Molecular Medicine Department, Sapienza University of Rome, 00189 Rome, Italy; † IRCCS Neuromed, 86077 Pozzilli, IS, Italy; ‡ Rehabilitation Department, A.O. “G. Salvini” 20024 Garbagnate Milanese, Milan, Italy

**Keywords:** bradykinin, heart failure, LCZ696 (sacubitril/valsartan), natriuretic peptides, neprilysin, neuroendocrine syndrome

## Abstract

After its discovery in the early 1980s, the natriuretic peptide (NP) system has been extensively characterized and its potential influence in the development and progression of heart failure (HF) has been investigated. HF is a syndrome characterized by the activation of different neurohormonal systems, predominantly the renin–angiotensin (Ang)–aldosterone system (RAAS) and the sympathetic nervous system (SNS), but also the NP system. Pharmacological interventions have been developed to counteract the neuroendocrine dysregulation, through the down modulation of RAAS with ACE (Ang-converting enzyme) inhibitors, ARBs (Ang receptor blockers) and mineralcorticoid antagonists and of SNS with β-blockers. In the last years, growing attention has been paid to the NP system. In the present review, we have summarized the current knowledge on the NP system, focusing on its role in HF and we provide an overview of the pharmacological attempts to modulate NP in HF: from the negative results of the study with neprilysin (NEP) inhibitors, alone or associated with an ACE inhibitor and vasopeptidase inhibitors, to the most recently and extremely encouraging results obtained with the new pharmacological class of Ang receptor and NEP inhibitor, currently defined ARNI (Ang receptor NEP inhibitor). Indeed, this new class of drugs to manage HF, supported by the recent results and a vast clinical development programme, may prompt a conceptual shift in the treatment of HF, moving from the inhibition of RAAS and SNS to a more integrated target to rebalance neurohormonal dysregulation in HF.

## Abstract (precis)

This article overviews the natriuretic peptides (NPs) system, its role and the development of NP-based treatment of heart failure (HF).

## NEUROHORMONAL ACTIVATION IN CHRONIC HEART FAILURE

Chronic heart failure (HF) is a syndrome with a complex pathophysiology in which the neuroendocrine activation plays a significant role. The pathophysiology of HF is indeed characterized by an early activation of different neurohormonal systems [i.e., sympathetic nervous system (SNS) and natriuretic peptides (NPs)] in the presence of asymptomatic left ventricular (LV) systolic dysfunction [1–4]. In the early stages of HF, the SNS and renin–angiotensin (Ang)–aldosterone system (RAAS) response play a compensatory role, aimed at supporting cardiac output and increasing peripheral vasoconstriction in an effort to maintain circulatory homoeostasis. However, the prolonged activation of the two systems becomes detrimental and contributes to progression and worsening of HF, eventually leading to congestion. In addition to the classical components of neuroendocrine activation other regulatory systems are involved, i.e. kinins, NPs, endothelin, erythropoietin, prostaglandins and adrenomedullin [5,6]. If the activation of SNS and RAAS results in unfavourable consequences and a negative prognostic affect, the activation of kinins and NP systems might play a favourable role.

**Figure 1:**
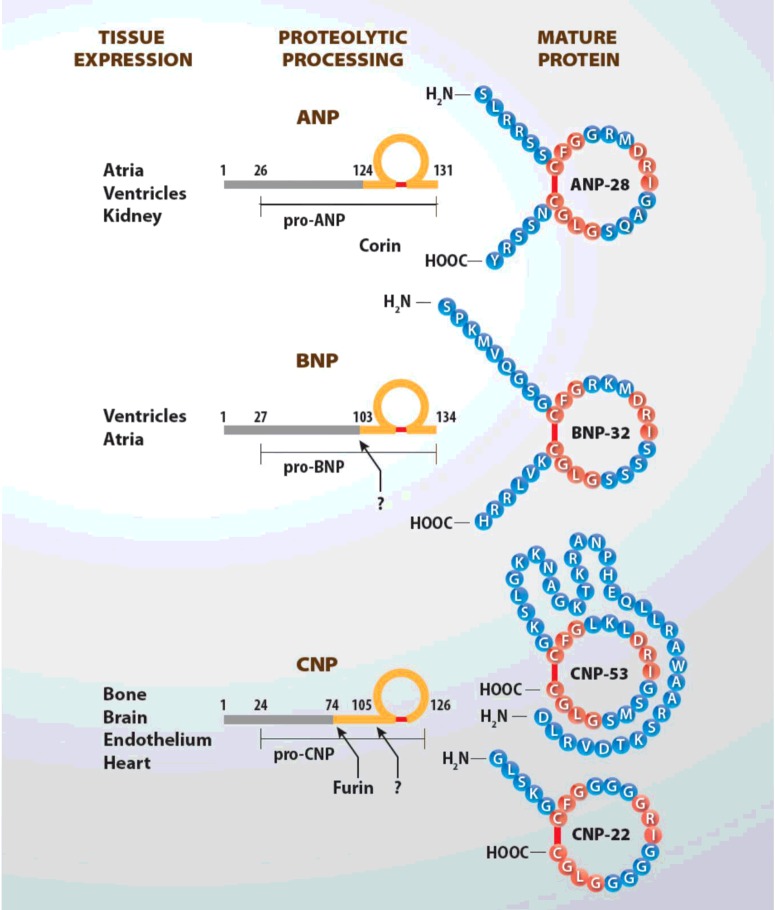
Tissue expression of NPs, proteolytic activation processing from pre-prohormones to mature and biologically active peptides

Therefore, pharmacological interventions aimed at re-balancing the neuroendocrine dysregulation in HF are effective and beneficial. So far, the therapeutic approach has been based on pharmacological interventions to down-modulate RAAS, through Ang-converting enzyme (ACE) inhibitors [7–12] or Ang receptor blockers (ARBs) [13–16], aldosterone [17–20] and also through mineralocorticoid receptor antagonists (MRA) and SNS by β-blockers [21–26]. Hence, triple-therapy with ACE inhibitors (or, if not tolerated, an ARB), β-blockers and MRA, currently represents the standard, optimal therapeutic approach (Figure 1). Triple-therapy improves symptoms and quality of life and provides protection against major fatal and non-fatal events, thus reducing hospitalization and mortality. If, for some reason, a patient cannot tolerate an MRA in addition to an ACE inhibitor and a β-blocker, an ARB can be tried as an alternative [15]. In addition to the triple-therapy, the treatment of HF include also vasodilators, as nitrates, and diuretics that are important to relieve signs and symptoms of congestion, inotropes and non-pharmacological tools such as implantable cardioverter-defibrillator, cardiac resynchronization therapy, mechanical ventricular assistance and heart transplantation [26].

## UNMET THERAPEUTIC NEEDS IN HEART FAILURE

In spite of an optimized therapy for the control of neuroendocrine activation and a consequent decline of hospital mortality [27], the quality of life and prognostic outlook for those HF patients who survive after an acute hospitalization remain poor [28]. Data suggest that HF-related survival rates are as poor as those associated with cancer. For example, in the original and subsequent Framingham cohort, 62% and 75% of men and 38% and 42% of women respectively, died within 5 years of being diagnosed with HF [29,30]. In comparison, 5-year survival for all cancers among men and women in the US during the same period was approximately 50%.

Therefore, in spite of the current approach to neuroendocrine modulation in HF, clinical goals remain largely unaccomplished and more thorough approaches to address neurohormonal dysregulation may be needed. From this point of view, the modulation of systems that promote favourable effects in HF, such as kinins and NP systems, has long been viewed as attractive and rational to implement benefits of RAAS and SNS blockade [31].

The block of bradykinin breakdown is potentially beneficial, because bradykinin has useful actions in HF [32]. Augmentation of bradykinin may promote vasodilation, fibrinolytic effects and inhibition of cellular growth and division, which may contribute to the benefits of ACE inhibitors [33–35]. An excess of bradykinin accumulation however may cause adverse effects, such as cough, rash, hypotension and angioedema [35]. A more promising strategy is represented by an empowerment of the NP system, which so far has not been effectively and safely attained in clinical practice.

## NATRIURETIC PEPTIDES SYSTEM

The cardiac endocrine function was first identified more than 30 years ago [36]. Since then, a growing number of experimental and clinical studies have been performed to investigate its components, regulation and function. Overall, many advances have been made in the field, consolidating the concept that the endocrine function of human heart is a relevant component of a complex network including endocrine, nervous and immune systems. In 1981, de Bold et al. [36] found that the intravenous injection of atrial, but not ventricular, homogenates into rats elicited a rapid decrease in blood pressure that was accompanied by increased renal sodium and water excretion. After this fundamental observation, several groups purified peptides of variable size from atrial tissue provided with both natriuretic and vascular smooth muscle-relaxing activity [37–40]. These peptides were collectively named atrial NP (ANP) or factor. B-type NP (BNP), which was originally called brain NP [41] and C-type NP (CNP) were subsequently purified from porcine brain extracts based on their ability to relax smooth muscle [42].

### Atrial natriuretic peptide

All NPs are synthesized as pre-prohormones (Figure 1). Human pre-proANP is 151-amino acids in length. Cleavage of the N-terminal signal sequence results in the 126-amino acid proANP, which is the predominant form stored in atrial granules. ProANP is rapidly cleaved upon secretion by the transmembrane cardiac serine protease to form the biologically-active C-terminal 28-amino acid peptide and the biologically inactive fragment (98 amino acid) of the ANP prohormone [N-terminal proANP (NT-proANP); 43]. Alternative processing of proANP by an unknown protease in the kidney generates a 32-residue peptide called urodilatin, which may be important in regulating renal sodium and water excretion [44].

ANP is primarily expressed and stored in granules in the atria, although it is present at lower concentrations in other tissues, such as the ventricles and kidney. The primary stimulus for ANP release is atrial wall stretch resulting from increased intravascular volume [45,46] or cardiac transmural pressure which may promote ANP expression and biosynthesis in the ventricles in conditions such as HF. Once secreted, ANP perfuses into the coronary sinus, which facilitates distribution to its various target organs in a true endocrine manner. Hormones such as endothelin [47], Ang [48] and arginine–vasopressin [49] stimulate ANP release [50].

Plasma levels of ANP in normal patients are approximately 10 fmol/ml (20 pg/ml) and are elevated 10–100-fold in patients with congestive HF [51,52]. Both ANP and NT-proANP have been used as markers for the diagnosis of asymptomatic LV dysfunction [53] and plasma ANP levels have been shown to correlate with the severity of symptomatic HF. However, in the last few years, due to higher stability, BNP measures were preferred to ANP for diagnostic and prognostic use in HF [54].

### B-type natriuretic peptide

BNP was initially purified from porcine brain extracts and hence defined brain NP [41]. However, it was subsequently found in much higher concentrations in cardiac ventricles from patients or animals undergoing cardiac stress such as congestive HF or myocardial infarction [52]. Therefore, it is currently referred to as BNP or B-type NP. Human BNP is synthesized as a pre-prohormone of 134 residues, containing a signal sequence that is cleaved to yield a 108-amino acid prohormone (Figure 1). The 108 amino acid precursor, proBNP, produced in cardiomyocytes, is cleaved between residues 76 and 77 by the processing enzymes corin or furin to produce the biologically-active 32-amino acid BNP plus the 76-amino acid N-terminal peptide [N-terminal proBNP (NT-proBNP)]. All three peptides proBNP, BNP and NT-proBNP are secreted by the heart and circulate in humans. Fully processed BNP length varies among species; human BNP is 32 amino acids [55].

Actually, the introduction of sensitive MS methods have led to the identification of many new low molecular mass circulating forms of BNP and the absence or near-absence of the 32-amino acid form in some subjects. In patients with HF, BNP(1–32) form is a minor constituent in peripheral plasma being rapidly truncated to BNP(3–32) upon incubation in plasma. This process is catalysed by the enzyme dipeptidyl peptidase IV (DPP IV), which rapidly removes the N-terminal serine-proline dipeptide from BNP. These truncated forms [BNP(3–32) or BNP(8–32)] elicit reduced natriuretic, diuretic and vasodilatory responses and increase urinary cGMP to a lesser extent than BNP(1–32), probably because of a more rapid degradation compared with BNP(1–32). DPP IV also cleaves a similar histidine-proline dipeptide off the N-terminal end of proBNP, although at a 4-fold slower rate, to produce the truncated proBNP(3–108) form. This peptide circulates at relatively low concentrations in normal subjects, but proBNP(3–108) concentrations are raised in patients with asymptomatic LV dysfunction or HF (fiftieth percentiles 8, 18 and 43 pmol/l respectively) [56].

Although BNP is stored with ANP in atrial granules, BNP is not stored in granules in the ventricles. Instead, ventricular BNP production is transcriptionally regulated by cardiac wall stretch resulting from volume overload or increased transmural gradient [57]. Healthy individuals have plasma BNP concentrations of approximately 1 fmol/ml (3.5 pg/ml). In patients with congestive HF plasma BNP concentration, as well as those of the inactive precursor NT-proBNP, increase up to 100-fold [53]. Both these peptides are commonly used as ‘rule-out’ test in the diagnosis of HF and as markers of prognosis in chronic HF. NT-proBNP concentrations in healthy individuals are approximately of 51 pg/ml, cut-off levels are 300 pg/ml for the diagnosis of acute HF and 125 pg/ml for chronic HF. For BNP, cut-off concentrations for the diagnosis of acute and chronic HF are respectively 100 and 35 pg/ml [26].

### C-type natriuretic peptide

CNP is the most widely expressed NP in the brain and is found in high concentrations in chondrocytes [58,59] and cytokine-exposed endothelial cells [60]. CNP is the most preserved form of NP: both 22- and 53-amino acid versions of CNP are identical in humans, pigs and rats. Human proCNP contains 103 residues and the intracellular endoprotease furin has been shown to process proCNP to the mature 53-amino acid peptide (Figure 1) [61]. In some tissues, CNP-53 is cleaved to CNP-22 by an unknown extracellular enzyme. Although CNP-22 and CNP-53 elicit similar, if not, identical functions [62], their tissue expression differs. CNP-53 is the major form in the brain [63], endothelial cells [64] and heart [65], whereas CNP-22 predominates in human plasma and cerebral spinal fluid [66]. Normal plasma CNP concentrations (both forms) are in the low fmol/ml range and are minimally, if at all, elevated in patients with congestive HF [67]. Therefore, CNP does not behave as a cardiac hormone, although its potential role in HF cannot be ruled out.

### Natriuretic peptide receptor

**Figure 2:**
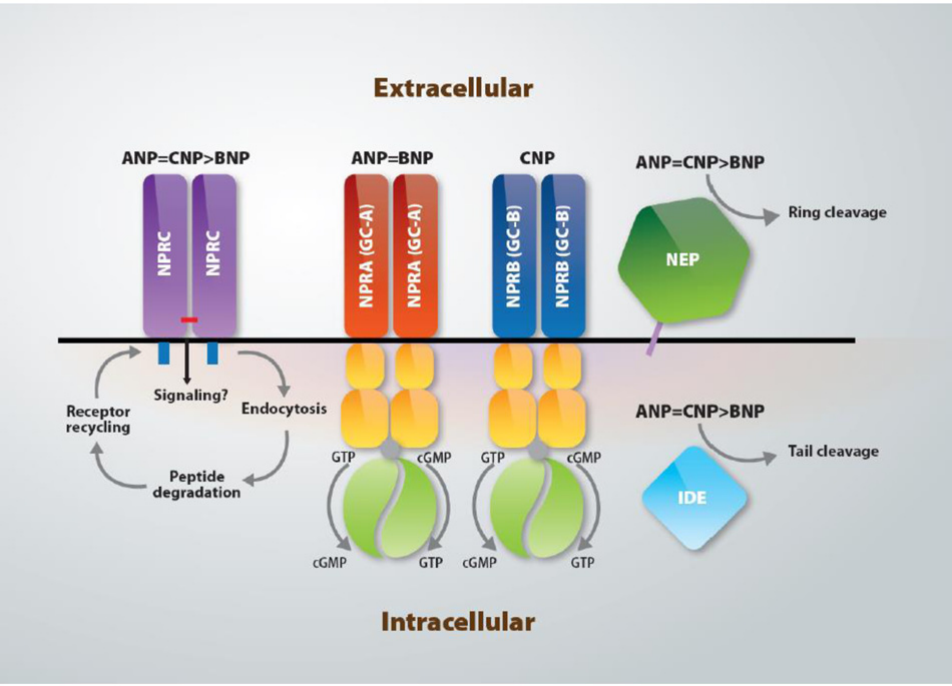
Natriuretic peptide-binding receptors, intracellular signalling and degradation processes Abbreviations: GC-A: guanylate cyclase type A; GC-B: guanylate cyclase type B.

There are three known NP-binding proteins in mammals: NP receptor (NPR)-A, NPR-B and NPR-C (Figure 2). NPR-A and NPR-B represent two of the five transmembrane guanylate cyclases found in humans [68] and determine the biological effects of NP. The third NPR, NPR-C, does not possess any known intrinsic enzymatic activity. Human NPR-A mRNA is highly expressed in kidney, adrenal, terminal ileum, adipose, aortic and lung tissues [69]. NPR-B protein has been found at relatively high concentrations in fibroblasts [70]. NP clearance receptor (NPR-C) mRNA is found in atrial, mesentery, placenta, lung, kidney, venous tissue [71] and in aortic smooth muscle and aortic endothelial cells. *In situ* hybridization studies found detectable NPR-C mRNA in kidney, adrenal, heart, cerebral cortex and cerebellum tissue [72].

### Physiological effects of natriuretic peptides

NPs elicit their physiological responses (Table 1) mostly through NPR-A binding and the activation of guanylate cyclase and the production of cGMP, a classic intracellular second messenger [73]. The best-studied cGMP signalling effects occur through protein kinases G (PKGs), serine and threonine kinases that are activated by cGMP binding [74].

**Table 1:** Physiological actions of NP Abbreviations: AVP, vasopressin; VSMC, vascular smooth muscle cells.

Target organ	Biological effects
Kidney	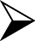 Increased GFR by inducing vasodilatation of afferent arterioles and vasoconstriction of efferent arterioles
	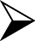 Induction of natriuresis by inhibiting Na^+^, H^+^ exchanger in the proximal tubule, Na^+^, Cl^−^ co-transporter in the distal tubule and Na^+^ channels in the collecting duct
	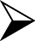 Induction of diuresis due to inhibition of AVP-induced acquaporin-2 incorporation into collecting ducts' apical membrane
Cardiac	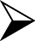 Reduction in preload leading to fall in cardiac output
	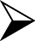 Inhibition of cardiac remodelling
Haemodynamic	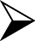 Vasorelaxation
	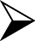 Elevating capillary hydraulic conductivity
	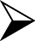 Decreased cardiac preload and afterload
Endocrine	Suppression of the following:
	- Renin–Ang–aldosterone axis
	- Sympathetic outflow
	- AVP
	- Endothelin
Mitogenesis	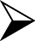 Inhibition of mitogenesis in VSMC
	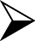 Inhibition of growth factor-mediated hypertrophy in fibroblasts

The ANP/NPR-A coupling regulates basal blood pressure in experimental models. Some of the most striking data on this issue come from the demonstration that ANP-dependent guanylate cyclase activities and blood pressure are directly proportional to NPR-A gene dosage over a range of 0–4 alleles [75]. ANP regulates blood pressure by means of its combined effects on intravascular volume, vasorelaxation, natriuresis and diuresis.

In the kidney, ANP increases glomerular filtration rate (GFR), inhibits sodium and water re-absorption and reduces renin secretion [76]. ANP-dependent diuresis and natriuresis are mediated exclusively by NPR-A in mice because these effects are completely lost in NPR-A knockout animals [77]. ANP increases the GFR by elevating the pressure in the glomerular capillaries through co-ordinated afferent arteriolar dilation and efferent arteriolar constriction [78]. In addition to these effects, ANP inhibits sodium and water re-absorption throughout the nephron. In the proximal tubules, ANP inhibits Ang II-stimulated sodium and water transport [79]. In collecting ducts, it reduces sodium absorption by inhibiting an amiloride-sensitive cation channel [80]. The effect of ANP on both transport processes is cGMP-dependent. ANP/NPR-A coupling appears to be down-regulated in HF and in conditions of RAAS activation and high Ang-II concentrations [81–83].

CNP also is a vasodilator and is released in response to vascular injury [84] NPR-B is present in aortic vascular smooth muscle and mediates CNP relaxation of pre-contracted rat aorta [85]. Furthermore, CNP inhibits vascular smooth muscle proliferation [86] and oxidized low-density lipoprotein-induced migration of cultured human coronary artery smooth muscle cells [87] in a cGMP-dependent manner. CNP may be an endothelium-derived hyperpolarizing factor (EDHF), participating in the paracrine action of other endothelial vasorelaxant mediators, such as nitric oxide (NO) and prostacyclin [88]. CNP may act also through a post receptor intracellular pathway linked to cAMP.

ANP regulates blood pressure, in part, through the inhibition of the RAAS. In dogs, intra-renal ANP infusion markedly inhibits the renin secretion rate [89,90]. In addition to inhibiting renin secretion, ANP directly inhibits aldosterone production in the adrenal glomerulosa (adrenocorticotropic hormone [ACTH]-stimulated, Ang-II-stimulated and basal aldosterone levels) [91,92]. Interestingly, ANP has also been shown to modulate arterial and cardiac baroreflex mechanisms in animal model and in humans. In particular, ANP enhances vagal afferents and blunts sympathetic response [93–95].

ANP and BNP have direct effects on the heart. Mice lacking ANP or NPR-A have enlarged hearts [96], whereas animals overexpressing ANP have smaller hearts [97]. Initially, it was unclear whether the cardiac hypertrophy observed in the knockout animals resulted from prolonged exposure to systemic hypertension or from the loss of a local inhibitory effect on heart growth; it is likely that both processes lead to cardiac hypertrophy. The first evidence supporting a local effect involved NPR-A knockout mice that were treated with anti-hypertensive drugs from birth. These animals were normotensive but still had cardiac hypertrophy [98]. The selective transgenic replacement of NPR-A in the heart of NPR-A knockout animals reduced cardiomyocyte size without affecting hypertension [99]. Pre-clinical data have demonstrated the ability of ANP to inhibit cardiomyocyte hypertrophy induced by either Ang-II or endothelin-1, both vasoactive peptides with deleterious effects on the cardio-renal system, as a result of cGMP-dependent processes [100]. Moreover, ANP may protect against Ang-II-induced cardiac remodelling by minimizing steps that are key to the inflammatory process including macrophage infiltration and expression of pro-inflammatory factors [101]. *In vitro* evidence indicates that ANP can attenuate norepinephrine-induced growth of cardiac myocytes and fibroblasts due to a cGMP-mediated inhibition of norepinephrine-induced influx of Ca^2+^ [102]. These findings may highlight a key role of the NP system in counteracting the adverse effects of increased SNS activity on the myocardium [94,95]. Finally, mutated forms of ANP are associated with cardiac hypertrophy [103].

All three NPRs are highly expressed in the lung [104]. ANP stimulates the dilation of pulmonary airways and blood vessels. Infusion or inhalation of ANP stimulates bronchodilation in normal and asthmatic patients [104]. ANP and BNP are elevated in patients with pulmonary hypertension and are indicative of increased right ventricular strain [105]. Mice overexpressing ANP are resistant to hypoxia-induced hypertension, whereas ANP-deficient mice exhibited increased pulmonary hypertension in response to chronic hypoxia [106]. CNP also reduces pulmonary hypertension [107] and fibrosis [108] and this mechanism is thought to be relevant in the progression of HF.

ANP stimulated lipolysis both in isolated human fat cells and in *in vivo* by peptide infusion [109]. It was determined that ANP-stimulated lipolysis is specific to primates, presumably because primates contain a higher NPR-A to NPR-C ratio [110]. PKGI is the cGMP effector in the ANP-dependent lipolytic response because pharmacological inhibition of PKGI decreases ANP-dependent lipolysis in primary human pre-adipocytes [111].

### Degradation of natriuretic peptides

All three NPs are degraded through two main processes (Figure 2): (1) NPR-C-mediated internalization followed by lysosomal degradation and (2) enzymatic degradation by neutral endopeptidase 24.11 or neprilysin (NEP), a zinc-dependent enzyme expressed on the plasma membrane that has broad substrate specificity and tissue distribution [112].

The reported half-life of ANP ranges from 0.5 to 4 min in mice, rats, rabbits, dogs and monkeys [113] and is approximately 2 min in normal human subjects [114,115]. Most tissues remove ANP from the circulation, but some organs are more efficient at ANP extraction than others. Early human studies indicated that approximately 30%–50% of ANP is removed by the kidney, liver or lower limbs, whereas no extraction was observed across the lung [116,117]. However, later reports in humans and dogs indicated that the lungs have a significant ANP extraction rate of between 19% and 24%. The organ preference for ANP extraction is lung > liver > kidney [118].

Few studies have addressed the clearance of BNP and CNP. The removal of BNP from the human circulation recognized short and long half-life components of 3.9 and 20.7 min respectively [52]. BNP binds to human NPRC 7% as tightly as ANP and the increased half-life of BNP results from decreased removal by NPRC-mediated internalization and degradation [119].

NPR-C-mediated ANP clearance was first demonstrated by Maack et al. in 1987 [120]. The cellular mechanics of NPRC-mediated NP internalization and degradation are similar to those of the receptors for low-density lipoprotein and hyaluronic acid. Similar features include lysosomal ligand hydrolysis and recycling of the ligand-free receptor back to the plasma membrane. Internalization is speculated to occur through a clathrin-dependent mechanism, but this has not been demonstrated. NPs are also degraded by extracellular proteases (Figure 2). NEP, the most important one, was initially discovered in rabbit kidney brush border membranes as a metalloenzyme that degrades the insulin β-chain [121] and subsequently as an enkephalinase and β-amyloid-degrading enzyme [122]. NEP is a zinc-containing, membrane-bound, ectoenzyme that cleaves substrates on the amino side of hydrophobic residues [121]. ANP-degrading activity in solubilized rat membranes co-purifies with NEP and is blocked by specific NEP inhibitors [123].

Purified NEP binds and degrades NPs similarly to other peptide hormones such as Ang-II [124]. Seven ANP cleavage sites were identified, but the initial attack occurs between Cys^7^ and Phe^8^, breaking the ring and inactivating the peptide [125]. NEP also efficiently cleaves CNP at multiple sites and, as with ANP, the initial cleavage site is between the conserved cysteine and the phenylalanine residues [126]. The ring structures of both ANP and CNP are essential for hydrolysis, because reduction and alkylation of the peptides greatly reduced degradation. In contrast with ANP or CNP, which have one or zero amino acid differences between human and rodent forms, BNP varies greatly among species [127]. Studies with purified enzymes indicated that BNP is a poorer substrate for human or porcine NEP than ANP or CNP. NEP cleaves human BNP at M^5^-V^6^ and R^17^-I^18^, but not at the conserved C^10^-F^11^ bond [128]. NEP-dependent degradation of BNP is species-specific. Although NEP accounts for most of the BNP-degrading activity in rat kidney membranes, NEP inhibitors failed to block BNP degradation by human kidney membranes, suggesting that NEP is not a significant regulator of BNP concentrations in the human kidney [129,130].

ANP is also cleaved by insulin-degrading enzyme (IDE), a zinc metalloprotease that is found in both cytoplasmic and membrane fractions and has diverse substrate specificity [131] (Figure 2). On the basis of competition with insulin for ANP degradation and a partial amino acid sequence of the 112-kDa protein, IDE was suggested to be an ANP-degrading enzyme [132]. Proteolysis of rat ANP, porcine BNP-26 and CNP with purified IDE revealed that ANP is the preferred substrate [133].

The relative contributions of NPR-C and NEP to ANP degradation have been investigated in a number of animal systems, with various NPR-C-blocking peptides and NEP inhibitors. Under normal conditions, infusion of NPR-C-blocking peptides has an effect on circulating ANP concentrations and associated physiological functions that is slightly greater than or equal to that of various NEP inhibitors [134–137]. However, in all cases examined, maximum ANP concentrations require inhibition of both degradation pathways. In pathological or pharmacological scenarios where NP concentrations are elevated and NPR-C may be saturated, NEP plays a more significant role in ANP degradation [138]. Both NPR-C and NEP pathways contribute to the degradation of BNP and CNP as well, although the exact contribution of each pathway to BNP concentrations is unclear [129]. NEP inhibition reduced CNP clearance by the kidney but not the lung, suggesting that NEP significantly contributes to CNP degradation in some but not all tissues.

## THE NATRIURETIC PEPTIDE SYSTEM IN HEART FAILURE

Congestive HF is a complex syndrome characterized by sodium and water retention. A decrease in cardiac output and effective intra-arterial volume leads to renal retention of sodium and water despite expansion of the extracellular fluid volume. This phenomenon occurs in the presence of a progressive activation of the NP system [139]. However, this response is apparently insufficient to counteract the activation of vasoconstriction and sodium retention of RAAS and SNS [140,141].

Several clinical and experimental studies have implicated both ANP and BNP in the pathophysiology of the deranged cardiorenal axis in HF. Early studies have shown that ANP release in response to acute volume overload or chronic salt loading are impaired in asymptomatic patients with cardiomyopathy and LV dysfunction [4,142]. This impairment of ANP adaptation to volume challenge in the early stages of HF is associated with blunted natriuretic, vasodilator and renin–aldosterone suppressing actions. Therefore, this hormonal abnormality may play a role in the progression of sodium retention and vasoconstriction in HF. It is interesting that pre-treatment with RAAS-inhibiting drugs, such as ACE inhibitors, may partially restore the ANP secretagogue response to volume expansion in HF [126] and that pre-treatment with an ARB may preserve the natriuretic and renal vasodilating capacity of ANP in the same condition [5].

Although initially considered to be a state of ANP deficiency, it soon became evident that in HF plasma levels of ANP are systematically elevated and positively correlated with the severity of the disease, as well as with the elevated atrial pressure and other parameters of LV dysfunction [143]. The highest concentrations of ANP in the circulation occur in advanced stages of HF and the high levels of plasma ANP are attributed to increased right-sided heart production rather than to decreased clearance. Although volume-induced atrial stretch is the main source for the elevated circulating ANP levels in HF, enhanced synthesis and release of the hormone by the ventricular tissue in response to Ang-II and endothelin contribute to this phenomenon [144,145]. Vagal stimulation linked to circulatory baroreflex control or to cardiac mechanoreceptor modulates ANP production and release from the heart [94,140,141]. As mentioned above, even the highest levels of circulating NPs due to massive cardiac secretion in advanced end stage congestive HF may indeed be insufficient to counteract the over-stimulation of the anti-natriuretic and vasoconstrictive neurohormonal system (SNS and RAAS) thus characterizing HF as a state of neurohormonal imbalance. Moreover, this has also been observed in models characterized by overstimulation of RAAS or high levels of Ang-II [146].

Despite the high levels of this potent natriuretic and diuretic agent, patients and experimental animal models with HF retain salt and water due to attenuated renal responsiveness to NP. In patients with HF [147] and in dogs with HF [148], ANP acts as a counter-regulatory hormone to SNS, RAAS and vasopressin. Infusion of pharmacological doses of synthetic ANP to experimental animals [149] and also to patients with HF [150] has consistently demonstrated an attenuated renal response compared with normal control subjects. This could be linked to a down-regulation of NP receptors in a condition of overstimulation of RAAS [81–83]. However, other beneficial effects accompany the infusion of ANP to patients with HF, such as haemodynamic improvement and inhibition of activated neurohumoral systems [151]. In fact, despite the blunted renal response to ANP in HF, elimination of this peptide by surgical means aggravates the activation of these vasoconstrictive hormones in this disease state. For instance, elimination of ANP source in dogs with HF due to rapid pacing by atrial appendectomy resulted in substantial increments in plasma renin activity and plasma norepinephrine, as well as marked sodium and water retention [152]. The activation of these systems was less profound in dogs with HF that did not undergo appendectomy, suggesting that ANP plays a critical role as a suppressor of sodium-retaining systems. Therefore, the increase in circulating NP, albeit insufficient to preserve sodium and water balance, is still considered an important adaptive or compensatory mechanism aimed at reducing peripheral vascular resistance and effective blood volume. The initial chronic phase of HF, which is characterized by sodium balance despite cardiac dysfunction, has been attributed to the elevated levels of ANP and BNP [153].

This notion is supported by the findings that inhibition of NPR in experimental HF induces sodium retention [154]. Furthermore, NPs inhibit the systemic vasoconstrictive effect of Ang-II [155], Ang-II-stimulated proximal tubule sodium re-absorption [156], Ang-II-enhanced secretion of aldosterone and the secretion of endothelin [157]. Therefore, NPs in HF act as an ideal counter-regulatory mechanism, influencing renal blood flow (RBF) and sodium excretion either through their direct renal actions or through inhibition of release or action of other vasoconstrictive agents. Moreover, beside these cardiovascular and endocrine effects, NPs probably play an important role in promoting salt and water excretion by the kidney in HF. Indeed, studies in an experimental model of HF have demonstrated that inhibition of the NP by either specific antibodies to their receptors or the NPR-A antagonist HS-142-1 causes further impairment in renal function, as expressed by increased renal vascular resistance and decreased GFR, RBF, urine flow, sodium excretion and activation of the RAAS [158].

Moreover, the unexpectedly small physiological responses to the apparently high levels of BNP previously observed in patients with HF may be because most of the BNP detected using conventional diagnostic assays is less biologically active. Consequently, HF may in fact represent a deficient state of biologically-active NP. The raised levels of biologically-inactive BNP detected in chronic HF may reflect a potential abnormality in the processing of NP, leading to a deficiency of mature BNP [159]. The increased expression of myocardial NEP mRNA in patients with HF, leading to an accelerated degradation of NPs, supports the hypothesis that a deficiency of NP may be present in HF [160].

The fact that the NP system plays a favourable role, counteracting the adverse effects of sodium-retaining and vasoconstrictive hormonal systems in HF, provides a rationale for the use of these peptides as therapeutic tool in this disease. Thus, either increasing the activity of the NP or reducing the influence of the anti-natriuretic systems by pharmacological means may achieve a favourable shift in the balance of sodium excretion and vasodilation in HF. In the interplay between the RAAS and ANP in HF, the approaches used in experimental and clinical medicine have included the decrease in the activity of the RAAS by means of ACE inhibitors, ARBs and MRA or increasing the activity of ANP or its second messenger, cGMP or a combination of both approaches.

## EXPLORING PHARMACOLOGICAL APPROACHES TO ENHANCE NATRIURETIC PEPTIDE EFFECTS IN HEART FAILURE

We discuss now previous pharmacological attempts to modulate NP in humans and, particularly, to develop new tools to exploit the theoretical advantages of raising NPs in HF.

### Exogenous natriuretic peptides and analogues

The first obvious strategy was based on exogenous NP or analogues administration. In fact, intravenous administration of ANP to patients with acute HF improved their clinical status [153]. Carperitide, a human recombinant form of ANP, was approved as an intravenous agent for the treatment of acute decompensated HF [127]. However, the short half-life restricted its routine use, prompting the development of novel forms of ANP that are more resistant to enzymatic degradation compared with both native and recombinant forms.

M-ANP is a 40-amino acid peptide consisting of the native 28-amino acid ANP with a 12-amino acid extension to the C-terminal. The extended C-terminal of M-ANP provides greater resistance, compared with native ANP, to NEP and IDE degradation and does not impair binding to NPR-A and cGMP generation [161]. *In vivo* studies in canines demonstrated that M-ANP possesses a greater blood pressure lowering effect than ANP. It also augments RBF and GFR (despite reductions in blood pressure), promotes natriuresis and diuresis and suppresses RAAS [161]. Recently, in a canine model of HF and acute vasoconstrictive hypertension, M-ANP was shown to possess an acute vasodilator effect similar to nitroglycerin; unlike nitroglycerin, M-ANP improves renal function through significant increases in RBF and GFR and inhibits aldosterone activation [162]. Based on these experimental results, M-ANP has now entered a clinical development programme for further testing.

Nesiritide, a recombinant form of human BNP, has been shown to decrease pulmonary capillary wedge pressure, to provide improvements in the global clinical status reducing dyspnoea and fatigue compared with placebo [163]. Nesiritide was approved for the treatment of acute decompensated HF in the USA in 2001 [127]. Subsequent reports of an increased risk of worsening renal function and death compared with control therapy raised doubts on its safety [164]. The Acute Study of Clinical Effectiveness of Nesiritide in Decompensated Heart Failure (ASCEND-HF) trial demonstrated that nesiritide had no impact on the rate of death, nor was it associated with worsening renal function [165]. However, nesiritide was associated with increased rates of hypotension. Together with its short bioavailability, this may represent a limitation for the routine use of the drug in clinical practice [165,166].

Cenderitide–NP (CD–NP), is formed by the fusion of native human CNP with a C-terminal sequence of dendroaspis NP (DNP) found in snake venom [167]. CD–NP is less susceptible to degradation by NEP than native NPs [168]. In a canine model, CD–NP elicited potent natriuretic and diuretic responses, increased GFR, inhibited renin and induced less hypotension than BNP. The anti-fibrotic actions of CD–NP have been demonstrated *in vivo* in an experimental rat model of early cardiac fibrosis [169]. In patients with chronic HF, subcutaneous infusion of CD–NP has previously been shown to provide a dose-dependent reduction in systolic blood pressure and to be well tolerated [170]. CD–NP is currently undergoing phase II clinical trials for chronic therapy in patients with post-acute HF. CU–NP, an alternative version of CD-NP, is constructed using a core component of native human CNP and the C- and N-terminal of urodilatin, a NP of renal origin that predominantly interacts with the NPR-A [171]. Preliminary *in vivo* data in a canine model have suggested that CU–NP may mediate beneficial cardiac and renal effects, reducing pulmonary capillary wedge pressure and right atrial pressure, inducing natriuresis, increasing GFR and suppressing RAAS [172].

### Neprilysin inhibition in treatment of heart failure

A second, more sound strategy is based on the pharmacological enhancement of endogenous NP level. Although this effect can be obtained by blocking clearance NPR receptors or by the inhibition of IDE, the pharmacological approach more frequently used in experimental models and in humans is represented by NEP inhibition.

In evaluating the clinical effects of NEP inhibition it is necessary to consider that in addition to the NP, NEP has many other substrates [173,174], with biological conflicting activity on vascular smooth muscle cell tone and renal excretion of sodium and water, including Ang-I [175], Ang-II [176], kinin peptides, substance P, adrenomedullin, endothelin, chemotactic peptide, enkephalins and the amyloid-β (Aβ) peptide [177]. NEP may also contribute to the formation of endothelin from its precursor big endothelin, although endothelin-converting enzyme (ECE) probably plays a more important role in endothelin formation [178].

**Figure 3:**
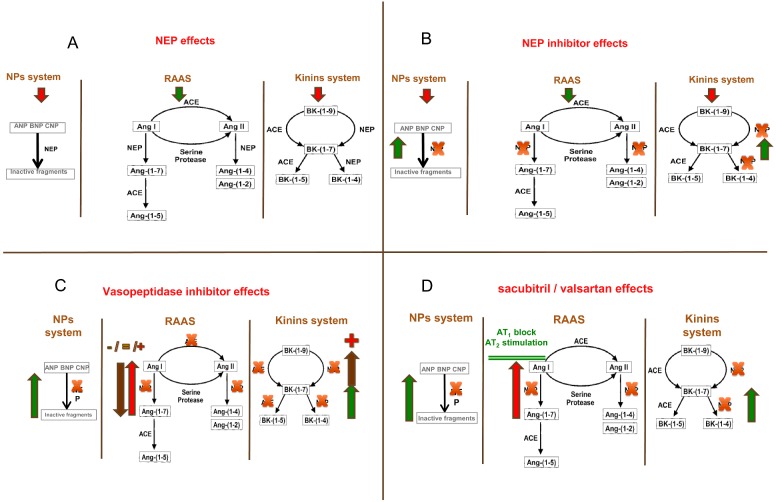
Effects of NEP activity on NP, RAAS and bradykinin system (**A**) and consequences of NEP inhibition (**B**), vasopeptidase inhibition (**C**) and ARNI (**D**) on the same systems **Abbreviation:** BK: bradykinin.

For patients with HF, of particular importance are the effects of NEP inhibition on RAAS and kinin peptides, systems that are also modulated by ACE-inhibitors (Figure 3A).

NEP inhibition may increase levels of the vasoconstrictor Ang-II and reduce levels of the vasodilator Ang-(1–7) [179]. In addition, both animal and clinical studies show that NEP inhibition impairs the metabolic clearance of Ang-II and increases plasma levels of Ang-I, Ang-II, aldosterone and catecholamines [180,181].

Moreover, NEP plays a major role in the metabolism of kinin peptides [182] and increased urinary kinin peptide levels may contribute to the natriuretic effects of NEP inhibition [183]. Kinins may also mediate the cardiac effects of NEP inhibition. NEP inhibition impairs kinin metabolism in the heart [181] and kinin receptor antagonism prevents the protective effects of NEP inhibition in animal models of ischaemia-reperfusion injury in the heart [184,185] and in isoproterenol-induced myocardial hypoperfusion [186]. Finally, since NEP is involved in the degradation of the Aβ peptides, which are implicated in the development of Alzheimer's disease, concerns have been raised that NEP inhibition might produce an accumulation of Aβ peptides in plaque-like deposits in the brain [187], although studies up to 5 years of follow-up have not supported this concern. Despite the favourable expectation related to the enhancement of the NP effects obtained through inhibition of NEP, clinical results obtained with multiple pharmacological intervention strategies used in the past have not prompted favourable clinical results in patient with HF, probably due to the concomitance of the effects that the inhibition of NEP induces on other systems [179], particularly RAAS and the kinin system.

#### Neprilysin inhibitors

The effects on blood pressure of a NEP inhibitor alone are quite variable. There may be no change, a decrease or an increase in blood pressure of normotensive and hypertensive human subjects in response to NEP inhibition [188–190].

The variable effect of NEP inhibition on blood pressure and systemic vascular resistance is likely to be related to the multiple actions of NEP on many different vasoactive peptides. Increased blood pressure during candoxatril treatment, a pure NEP inhibitor, in healthy volunteers was associated with an increase in plasma Ang-II and endothelin levels [191]. In hypertensive patients candoxatril did not produce clinically favourable effects [192] probably because the negative haemodynamic effects linked to activation of RAAS counteracted the positive effects related to raising NP (Figure 3B). Also, in patients with HF, the haemodynamic effects of candoxatril were not favourable, with an increase in peripheral vascular resistance and a stroke volume decrease [193].

As a result of neutral or negative clinical data obtained in hypertensive patients and, in HF, the clinical use of a NEP inhibitor alone was discouraged.

Taking together, the overall complex biological effects due to NEP inhibition and the unfavourable results obtained in the past, it was reasonable to try different strategies, such as NEP inhibitors, NEP inhibitors associated with an ACE inhibitor or vasopeptidase inhibitors.

#### Neprilysin inhibitors associated with an angiotensin-converting enzyme inhibitor

Addition of NEP inhibition to ACE inhibition produced indeed greater inhibition of bradykinin metabolism and higher bradykinin levels than those seen with either ACE or NEP inhibition alone [176]. The increased kinin peptide levels may contribute to the natriuretic, hypotensive and cardioprotective effects of addition of NEP inhibition to ACE inhibition. However, given the recognized role of kinin peptides in ACE inhibitor-induced angioedema [194,195] the additional downgrading of kinin metabolism by NEP inhibition would be predicted to increase the occurrence of angioedema. In addition, by increasing Ang-II levels and reducing Ang-(1–7) levels NEP inhibition may counteract any benefit of ACE inhibition that depends on reduced Ang-II levels and increased Ang-(1–7) levels.

Despite the potential advantages of the association between ACE inhibitor and NEP inhibitor, the clinical results obtained have not confirmed any usefulness of this therapeutic approach.

In particular, for patients with HF, the addition of the NEP inhibitor ecadotril to standard therapy, including ACE inhibition, produced no evidence of improvement in signs or symptoms [196]. Of particular concern was the occurrence of aplastic anaemia in several patients, which was attributed to the thioester group on the ecadotril molecule [197]. As a result of non-positive clinical data obtained, also this pharmacological option was abandoned.

#### Vasopeptidase inhibitors

Several dual ACE/NEP inhibiting molecules have been developed [198–200]. In assessing the results obtained with the so called vasopeptidase inhibition it is important to consider that the separate titration of ACE and NEP inhibition is not possible when both are mediated by a single molecule because the ratio of ACE to NEP inhibition is fixed (Figure 3C). It is therefore important to assess whether dual ACE/NEP inhibitors have therapeutic effects different from ACE inhibition alone.

The most extensively investigated dual ACE/NEP inhibitor was omapatrilat (BMS-186716). The initial experience with this compound was very promising, due to its potent blood pressure lowering effect. Then omapatrilat was tested in HF. In the Omapatrilat Versus Enalapril Randomized Trial of Utility in Reducing Events (OVERTURE) study 5770 patients with New York Heart Association (NYHA class II–IV HF were randomized to treatment with either enalapril [10 mg twice daily (BID)] or omapatrilat (40 mg once daily) for a mean of 14.6 months [201]. Enalapril or omapatrilat were added to conventional therapy that included β-blockers in 50% of patients. The primary end-point of combined risk of death or hospitalization for HF requiring intravenous treatment was not different for the two treatment groups. A reasonable explanation for the failure of omapatrilat to produce obvious advantages compared with ACE inhibitor was the short duration of NEP inhibition produced by this compound [202]. Although it may be argued that a higher dosage or BID dosage may have increased the benefits obtained with omapatrilat in HF, it could also be claimed that a higher dosage of enalapril may similarly increase the benefits from ACE inhibition alone. Angioedema was reported in similar proportions of omapatrilat-treated (0.8%) and enalapril-treated patients (0.5%). The slightly higher incidence of angioedema especially in the Afro-American population raised concerns and was shown later to be more relevant in hypertensive patients. In the OCTAVE study [203], angioedema was reported in 2.2% of patients receiving omapatrilat and in 0.7% of patients receiving enalapril. The high rate of angioedema in hypertensive patients and the non-superiority compared with ACE inhibitor in patients with HF led to the interruption of the clinical development of omapatrilat.

#### Angiotensin receptor neprilysin inhibition and ARB combination with LCZ696 (sacubitril/valsartan) compound

Quite recently, a novel pharmacological approach based on the combination of Ang receptor NEP inhibitor (ARNI) has been developed with the rational to generate a novel tool to target NP enhancement and RAAS blockade without increasing side effects, thus overcoming the drawbacks and failures encountered with the strategies described above (Figure 3D).

LCZ696, a first-in-class ARNI, comprises molecular moieties of valsartan, a well-established ARB [204] and of the NEP inhibitor prodrug sacubitril (AHU377), which is metabolized to the active NEP inhibitor LBQ657 by enzymatic cleavage of its ethyl ester [205]. LCZ696 is a novel single molecule in which the molecular moieties of valsartan and the molecular moieties of AHU377 are present in a 1:1 molar ratio. Pre-clinical studies in rats and dogs, performed by the producer and reported in the Investigator Brochure, demonstrated that inhibition of NEP enzyme activity and the AT1 (Ang II receptor type 1) receptor through LCZ696 increase the levels and effects of ANP while blocking the actions of Ang-II. This produces dose-related and long-lasting vasodilatory effects after single and multiple oral administrations, along with beneficial and protective effects on renal, vascular and cardiac tissue in rat models of hypertension, myocardial infarction and organ injury.

The bioavailability studies demonstrated that systemic exposure to valsartan following a single 400 mg of oral dose of LCZ696 was equivalent to that following administration of 320 mg of valsartan, a dose that has proven anti-hypertensive efficacy in clinical trials [166]. Analysis of dose-normalized pharmacokinetic data from either pre-clinical or clinical studies showed that the exposure of valsartan following administration of LCZ696 was approximately 40% higher than the exposure following administration of valsartan alone.

Inhibition of NEP activity increases levels of ANP, which in turn stimulates the synthesis of cGMP via guanylate cyclase-linked receptors. Pre-clinical pharmacodynamic studies demonstrated a rapid and dose-dependent increase in plasma ANP immunoreactivity following oral administration of LCZ696, whereas multiple-dose administration of LCZ696 in the dose escalation study in healthy participants significantly increased plasma cGMP levels [206]. These findings are consistent with inhibition of NEP activity.

LCZ696 stimulated significant dose-dependent increases in renin concentration, plasma renin activity and Ang-II concentration compared with placebo, indicative of blockade of the AT1 receptor by LCZ696. Notably, LCZ696 (200 mg) increased renin concentration by 3.1-fold, plasma renin activity by 4.9-fold and Ang-II by 3.7-fold relative to placebo. These increases are of a similar order of magnitude to those observed previously with administration of valsartan 320 mg in healthy participants not receiving a low-sodium diet [207,208]. Significant increases in all RAAS biomarkers were sustained 24 h after LCZ696 administration, consistent with the observed long plasma half-life of valsartan (15–22 h).

Pharmacokinetic data from the clinical dose escalation study showed that the peak concentrations of valsartan and LBQ657 were reached at about the same time following both single- and multiple-dose LCZ696 administration (1.5–4.5 h). This is reflected in the similar time-frame observed for pharmacodynamic effects, with the peak concentrations for both cGMP and RAAS biomarkers reached within 4 h after dosing with multiple-dose administration.

**Figure 4:**
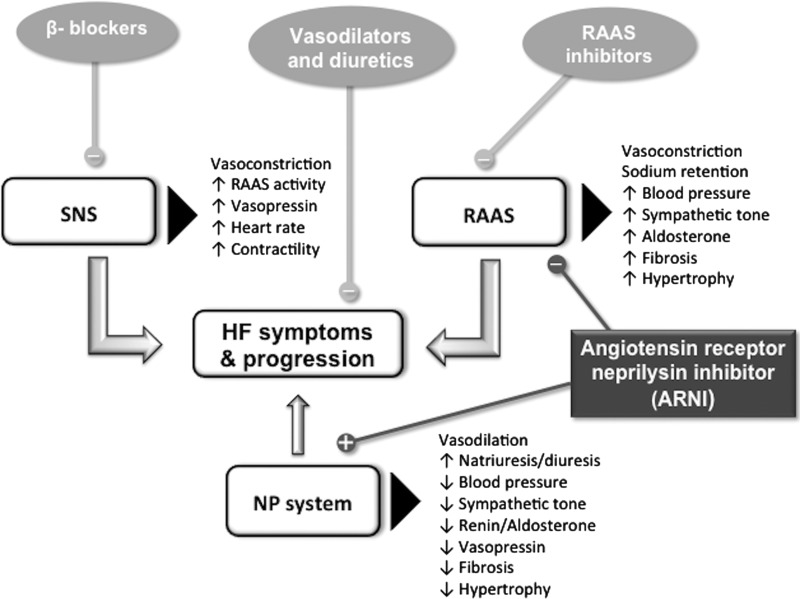
Current therapeutic approaches for HF and the novel role of ARNI

The concurrent effects of LCZ696 on NEP inhibition and AT1 receptor blockade may have synergistic benefits for clinical efficacy in hypertensive patients and also in patients with HF (Figure 4).

The Prospective comparison of ARNI with ARB on Management Of heart failUre with preserved ejectioN fracTion (PARAMOUNT) trial was a phase II study that evaluated the efficacy and safety profile of LCZ696 compared with valsartan in patients with chronic HF and preserved ejection fraction (HFpEF) [209]. NT-proBNP, a marker of LV wall stress associated with adverse outcomes in patients with HFpEF, was significantly reduced from baseline by LCZ696 compared with valsartan. The study also assessed the effect of LCZ696 on left atrial structure and function by measuring left atrial dimension, volume and volume index. An enlarged left atrium is a characteristic finding in patients with HFpEF and it reflects sustained increases in LV filling pressures. These parameters were significantly reduced from baseline to a greater extent in patients treated with LCZ696 compared with those treated with valsartan, thus indicating reverse left atrial remodelling. The tolerability profile of LCZ696 was satisfactory and similar to that of valsartan.

The effect of LCZ696 on outcomes compared with enalapril in patients with HFrEF (heart failure with reduced ejection fraction) was evaluated in the Prospective comparison of ARNI with ACEI to Determine Impact on Global Mortality and morbidity in Heart Failure (PARADIGM-HF) trial [210]. Eligibility requirements at screening included an age of at least 18 years, NYHA class II, III or IV and an ejection fraction of 35% or less. Patients were required to have a plasma BNP level of at least 150 pg/ml (or an NT-proBNP level ≥ 600 pg/ml) or, if they had been hospitalized for HF within the previous 12 months, a BNP of at least 100 pg/ml (or an NT-proBNP ≥ 400 pg/ml). Patients taking any dose of an ACE inhibitor or ARB were considered for participation, but for at least 4 weeks before screening, patients were required to take a stable dose of a β-blocker and an ACE inhibitor (or ARB) equivalent to at least 10 mg of enalapril daily.

Patients eligible were randomly assigned in a 1:1 ratio to double-blind treatment with either enalapril (at a dose of 10 mg BID) or LCZ696 (at a dose of 200 mg BID)

The primary outcome was a composite of death from cardiovascular causes or a first hospitalization for HF. The secondary outcomes were the time to death from any cause, the change from baseline to 8 months in the clinical summary score on the Kansas City Cardiomyopathy Questionnaire (KCCQ) [211], the time to a new onset of atrial fibrillation and the time to the first occurrence of a decline in renal function.

Patients (4187) were randomly assigned to receive LCZ696 and 4212 to receive enalapril for the intention-to-treat analysis. Death from cardiovascular causes or hospitalization for HF (the primary end point) occurred in 914 patients (21.8%) in the LCZ696 group and 1117 patients (26.5%) in the enalapril group [hazard ratio in the LCZ696 group, 0.80; 95% confidence interval (CI), 0.73–0.87; *P*<0.001 (exact *P*=4.0×10^−7^)]. The difference in favour of LCZ696 was seen early in the trial and at each interim analysis. A total of 558 deaths (13.3%) in the LCZ696 group and 693 (16.5%) in the enalapril group were due to cardiovascular causes (hazard ratio, 0.80; 95% CI, 0.71–0.89; *P*<0.001). Of the patients receiving LCZ696, 537 (12.8%) were hospitalized for HF, as compared with 658 patients (15.6%) receiving enalapril (hazard ratio, 0.79; 95% CI, 0.71–0.89; *P*<0.001).

A total of 711 patients (17.0%) in the LCZ696 group and 835 patients (19.8%) in the enalapril group died (hazard ratio for death from any cause, 0.84; 95% CI, 0.76–0.93; *P*<0.001). The effect of LCZ696 was consistent across all pre-specified representative population sub-groups.

The mean change from baseline to month 8 in the KCCQ clinical summary score was a reduction of 2.99 points in the LCZ696 group and a reduction of 4.63 points in the enalapril group (between-group difference, 1.64 points; 95% CI, 0.63–2.65; *P*=0.001).

Patients in the LCZ696 group were more likely than those in the enalapril group to have symptomatic hypotension, but these events rarely required the discontinuation of treatment. In contrast, cough, a serum creatinine level of 2.5 mg/dl (221 μmol/l) or more and a serum potassium level of more than 6.0 mmol/l were reported less frequently in the LCZ696 group than in the enalapril group (*P*<0.05 for all comparisons). There were no statistically significant differences between LCZ696 and enalapril in the incidence of angioedema (global non-hospitalized plus hospitalized 0.4% compared with 0.3% respectively) and, in particular, there were no cases in either group which resulted in airway compromise. Fewer patients in the LCZ696 group than in the enalapril group stopped their study medication because of an adverse event (10.7% compared with 12.3%, *P*=0.03) or because of renal impairment (0.7% compared with 1.4%, *P*=0.002).

**Figure 5:**
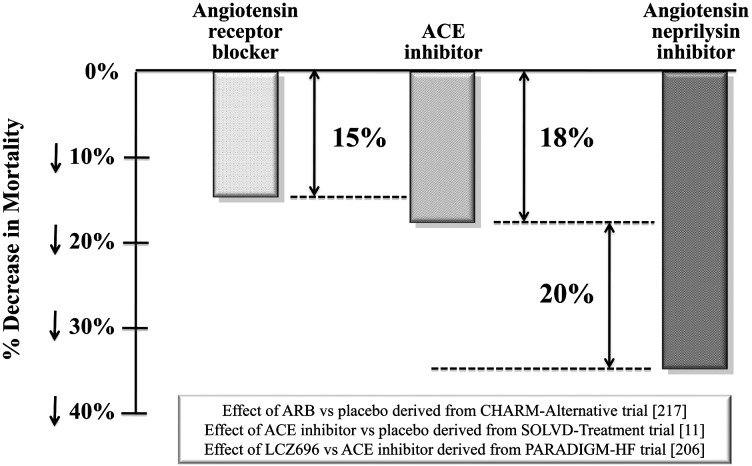
Ang NEP inhibition with LCZ696 doubles beneficial effect on cardiovascular death of current inhibitors of the renin–Ang system

In conclusion, LCZ696 was superior to ACE inhibition at recommended doses in reducing the risks of death and of hospitalization for HF. The magnitude of the beneficial effect of LCZ696, as compared with enalapril on cardiovascular mortality, was at least as large as that of long-term treatment with enalapril, as compared with placebo (Figure 5). According to a secondary analysis, LCZ696 prevented the clinical deterioration or progression of surviving patients in terms of required treatment intensification of therapy, hospital visits or admissions and use of advanced management modalities (inotropes, assist devices, transplantation) more effectively than did enalapril. Moreover, levels of both urinary cGMP and plasma BNP were higher during treatment with LCZ696 than with enalapril, reflecting the fact that the peptides, whose levels are enhanced by NEP inhibition, are active and bind receptors leading to enhancement of cGMP. In contrast, in comparison with enalapril, patients receiving LCZ696 had consistently lower levels of NT-proBNP (reflecting reduced cardiac wall stress) throughout the trial. The divergent effects of LCZ696 on the two types of NPs can be explained by the fact that BNP (but not NT-proBNP) is a substrate for NEP; thus, levels of BNP will reflect the action of the drug, whereas levels of NT-proBNP will reflect the effects of the drug on the heart [212] On the basis of these results, LCZ969 has been recently approved by the Food and Drug Administration (FDA) for the treatment of HFrEF; trade name is Entresto (http://www.fda.gov/NewsEvents/Newsroom/PressAnnouncements/ucm453845.htm).

The potential clinical relevance of these data is evident and these results may represent a new standard of treatment threshold for patients with HF [213]. However the extent of clinical benefits obtained with LCZ696, in many ways unexpected, at least for the observed size, poses questions about the biological plausibility of the results, which must be answered also in relation to previous experiences of pharmacological modulation of the NP system in patients with HF.

In spite of the obvious biological plausibility of the effects of LCZ969 on the NP system and RAAS in HF to explain the beneficial clinical findings, further work will be required to investigate these aspects in deeper detail. Among the various potential mechanisms that have been discussed after the publication of PARADIGM-HF, it seems unlikely, based on the previous clinical comparison between ACE inhibitors and ARBs that the benefits of LCZ696 are due to the ARB component of the ARNI by itself. In fact, in ELITE (Evaluation of Losartan in the Elderly study), VALIANT (VALsartan In Acute myocardial iNfarcTion), VAL-HEFT (Valsartan Heart Failure Trial) and CHARM (Candesartan in Heart failure - Assessment of moRtality and Morbidity) [14,214–217], ARB were never superior to ACE inhibitors. For similar reasons, a fundamental role of the NEP inhibitor acting as a diuretic added to the ARB can be excluded. The role of increased NP levels in the blood by itself also does not seem to represent a reasonable mechanism, in view of the negative results on outcomes of NEP inhibitors and vasopeptidase inhibitors. Nonetheless, it may be important that the presence of higher levels of NP are able to bind NPR-A receptors and to produce the biological response. In this regard, a role could also be played by a greater availability of NPR-A to be coupled by higher active NP and activate the guanylate cyclase pathway with a higher production of cGMP, which appears indeed to be the case in a sub-population of the PARADIGM-HF in which urinary cGMP was measured and indeed it was selectively increased in the group treated with LCZ696 [218]. The concomitant blockade of RAAS by the ARB valsartan may have partially antagonized the NPR-A down-regulation described in HF, given the interactions between the Ang-II and NP intracellular signalling pathways [219–222]. Finally, a role of the blood pressure lowering effect of LCZ696 cannot be completely ruled out, which may unload the heart and produce clinical benefits. In this regard, the blockade of the AT-1 receptor, which counteracts the increase in Ang-II due to NEP inhibition, by the concomitant ARB may play a relevant role.

Further studies are ongoing to analyse the clinical effects of LCZ696 in patients with HFrEF and HFpEF. The Efficacy and Safety of LCZ696 Compared to Valsartan, on Morbidity and Mortality in Heart Failure Patients With Preserved Ejection Fraction (PARAGON-HF) is a multicentre, randomized, double-blind, controlled clinical study with the aim to evaluate the effect of LCZ696 compared with valsartan in reducing the rate of the composite end-point of CV death and total (first and recurrent) HF hospitalizations in patients with HFpEF and NYHA class II–IV. Secondary outcomes are cumulative number of events of the extended composite end-point of CV death, total HF hospitalizations, total non-fatal strokes and total non-fatal myocardial infarctions, change from baseline to month 8 in NYHA functional class, time to new onset of atrial fibrillation and time to all-cause mortality (https://clinicaltrials.gov/ct2/show/NCT01920711).

In addition, LCZ696 has been investigated in comparison with olmesartan in patients with resistant hypertension, focusing on the effects on central blood pressure (PARAMETER study) [223].

## CONCLUSION

HF is a syndrome characterized by the activation of different neurohormonal systems such as SNS, RAAS and NP. So far, the therapeutic approach has been based on pharmacological interventions to down-modulate RAAS, through ACE inhibitors [193,196,201], ARBs and MRA and SNS through β-blockers. In the last years, more attention has been paid to potential advantages linked to the system of NP.

After the negative results of the study with NEP inhibitors, alone or associated with an ACE inhibitor and vasopeptidase inhibitors [193,196,201], recently, extremely encouraging results have been obtained with the new pharmacological class of Ang receptor and NEP inhibitor, currently defined ARNI. Indeed, LCZ696 produced a remarkable reduction in morbidity and mortality compared with optimal treatment of HF including enalapril up to a degree similar to that determined by the ACE inhibitors when compared with placebo. The new pharmacological approach to manage HF, supported by the results of PARADIGM-HF, may prompt a conceptual shift in the treatment of HF, moving from the inhibition of RAAS and SNS to the targeting of a neuro-hormonal re-balancing in HF. Further studies are ongoing to analyse the clinical effects of LCZ696 in patients with HFrEF and in patients with HFpEF (PARAGON-HF).

## Abbreviations

Aβ: amyloid-β
ACE: angiotensin-converting enzyme
Ang: angiotensin
ARB: angiotensin receptor blocker
ARNI: angiotensin receptor neprilysin inhibitor
ANP: atrial natriuretic peptide
AT1: angiotensin II receptor type 1
BID: twice daily
BNP: B-type natriuretic peptide
CD: cenderitide
CI: confidence interval
CNP: C-type natriuretic peptide
GFR: glomerular filtration rate
HF: heart failure
HFpEF: heart failure with preserved ejection fraction
HFrEF: heart failure with reduced ejection fraction
IDE: insulin-degrading enzyme
KCCQ: Kansas City Cardiomyopathy Questionnaire
LV: left ventricular
MRA: mineralocorticoid receptor antagonists
MS: mass spectometry
NEP: neprilysin
NP: natriuretic peptide
NPR: natriuretic peptide receptor
NT-proANP: N-terminal proANP
NT-proBNP: N-terminal proBNP
NYHA: New York Heart Association
PARADIGM-HF: Prospective comparison of ARNI with ACEI to Determine Impact on Global Mortality and morbidity in Heart Failure
PARAGON-HF: Efficacy and Safety of LCZ696 Compared to Valsartan, on Morbidity and Mortality in Heart Failure Patients With Preserved Ejection Fraction
PKG: protein kinases G
RAAS: renin–angiotensin–aldosterone system
RBF: renal blood flow
SNS: sympathetic nervous system
